# Clinical Characteristics, Microbiological Sources, and Outcomes of *Candida*-Positive ICU Cultures in Critically Ill Adults

**DOI:** 10.3390/jcm15103710

**Published:** 2026-05-12

**Authors:** Erdem Yalçınkaya, Umut Sabri Kasapoğlu, Hüseyin Arıkan, Ozan Çakmak, Şimal Beril Babaoğlu, Bilge İnce, Dilanur Salta, Zeynep Gökşin Canbir, Semiha Emel Eryüksel, Sait Karakurt

**Affiliations:** Department of Pulmonary and Critical Care Medicine, Marmara University School of Medicine, Istanbul 34854, Türkiye; umutkasapoglu@gmail.com (U.S.K.); arikanhuseyin@gmail.com (H.A.); cakmak.ozan@hotmail.com (O.Ç.); simalbabaoglu@gmail.com (Ş.B.B.); bilgeince2001@gmail.com (B.İ.); dilanursaltaa@gmail.com (D.S.); zeynepgoksincan@gmail.com (Z.G.C.); dreryuksel@gmail.com (S.E.E.); saitkarakurt@hotmail.com (S.K.)

**Keywords:** *Candida*-positive ICU cultures, intensive care unit, critical care, *Candida* score, candidemia, colonization, *Candida* infection, *Candida auris*, antifungal treatment

## Abstract

**Background:** *Candida* isolation is common in critically ill patients, but its clinical interpretation depends strongly on microbiological source, host factors, and clinical context. Bloodstream isolation, candiduria, respiratory tract isolation, surveillance cultures, catheter-tip cultures, and wound/skin cultures have different clinical implications. We aimed to evaluate clinical characteristics, microbiological sources, species distribution, antifungal treatment patterns, and outcomes among adult ICU patients with *Candida*-positive ICU cultures. **Methods:** This single-center retrospective observational cohort study was conducted in the medical intensive care unit of Marmara University Faculty of Medicine between 1 October 2022 and 5 September 2025. Adult ICU patients with at least one *Candida*-positive ICU culture were included. Non-*Candida* fungal isolates and duplicate patient-level records were excluded. The primary outcome was all-cause 28-day mortality. ICU mortality was defined as all-cause death during ICU stay. Source-stratified analyses and expanded multivariable logistic regression models were performed to evaluate factors associated with mortality. **Results:** A total of 349 adult ICU patients were included. Median age was 71 years [IQR, 62–82], and 185 patients were male (53.0%). Overall, 28-day mortality was 59.0% (206/349), and ICU mortality was 65.9% (230/349). *Candida* colonization was identified in 247 patients (70.8%), whereas *Candida* infection was identified in 102 patients (29.2%). The most common species were *Candida albicans* (48.4%), *Candida glabrata* (13.8%), and *Candida auris* (12.9%). The most frequent microbiological sources were urine (42.4%), lower respiratory tract samples (26.4%), and blood cultures (14.9%). Blood/sterile-site isolation was associated with higher ICU mortality than non-blood/non-sterile-site isolation (79.2% vs. 63.5%, *p* = 0.026), whereas the difference in 28-day mortality was not statistically significant (66.0% vs. 57.8%, *p* = 0.260). Antifungal treatment was more frequent among patients with blood/sterile-site isolation (94.3% vs. 16.9%, *p* < 0.001). In the expanded 28-day mortality model, lactate, NLR, and carbapenem exposure were independently associated with mortality. In the expanded ICU mortality model, lactate and CRRT/hemodialysis were independently associated with mortality. *Candida* score was not independently associated with either 28-day mortality or ICU mortality after broader adjustment. **Conclusions:** *Candida*-positive ICU cultures represent a heterogeneous clinical and microbiological spectrum. Source-specific interpretation is essential, particularly when distinguishing bloodstream or sterile-site isolation from non-sterile-site colonization. *Candida* score may reflect a higher-risk clinical phenotype, but it should not be interpreted as a stand-alone mortality prediction tool.

## 1. Introduction

Fungal infections remain an important cause of morbidity and mortality in critically ill patients, particularly among those exposed to broad-spectrum antibiotics, invasive devices, renal replacement therapy, total parenteral nutrition, corticosteroids, and prolonged intensive care unit (ICU) support [1,2,3,4]. Among ICU-associated fungal diseases, invasive candidiasis is the most frequent entity and includes candidemia and deep-seated candidiasis, both of which are associated with diagnostic uncertainty, delayed recognition, increased resource utilization, and poor clinical outcomes [1,2,5,6].

The burden of invasive candidiasis in the ICU remains substantial. In the EUCANDICU project, which evaluated ICU-acquired invasive candidiasis across European centers, the cumulative incidence was 7.07 cases per 1000 ICU admissions, and crude 30-day mortality was 42% [5]. Similarly, Al-Dorzi et al. reported high mortality among critically ill patients with invasive candidiasis in a prospective cohort from tertiary-care ICUs [6]. These findings support the need for improved interpretation of *Candida* isolation in ICU practice, particularly because microbiological positivity may represent a spectrum ranging from colonization to proven invasive disease.

A major challenge is that *Candida*-positive cultures from different anatomical and microbiological sources do not carry the same clinical significance. Bloodstream isolation of *Candida* spp. is generally interpreted as candidemia and requires prompt systemic treatment, whereas *Candida* isolation from respiratory secretions usually reflects airway colonization rather than true *Candida* pneumonia [1]. Similarly, candiduria frequently represents colonization, especially in catheterized or critically ill patients, and should not automatically be interpreted as invasive infection in the absence of compatible clinical features or high-risk conditions [1]. Therefore, pooled evaluation of candidemia, candiduria, respiratory tract isolation, surveillance cultures, catheter-tip cultures, and wound/skin cultures may obscure clinically meaningful differences unless source-specific analyses are performed.

This interpretive difficulty has led to increasing emphasis on standardized definitions and source-based classification. The recent FUNDICU 2024 consensus definitions were developed to improve the classification of invasive fungal diseases in non-neutropenic adult ICU patients and highlighted the importance of microbiological certainty, source of isolation, and clinical context when defining invasive fungal disease in this population [7]. Such frameworks are particularly relevant for retrospective ICU cohorts, in which prospective adjudication of infection versus colonization is often not feasible and microbiological data must be interpreted together with clinical severity, treatment exposure, and organ support variables.

Risk stratification tools have also been proposed to guide antifungal decision-making in critically ill patients. The *Candida* score described by León et al. was developed as a bedside tool to identify non-neutropenic critically ill patients with *Candida* colonization who may be at increased risk of invasive candidiasis and may require early antifungal treatment [8]. However, this score was not originally designed as a mortality prediction tool. Moreover, the EMPIRICUS randomized clinical trial showed that empirical micafungin in critically ill patients with ICU-acquired sepsis, multiple-site *Candida* colonization, and multiple organ failure did not improve 28-day fungal infection-free survival, despite reducing the occurrence of new invasive fungal infections [9]. These findings underscore the difficulty of translating colonization-based risk stratification into improved clinical outcomes and highlight the need for careful interpretation of *Candida* score in outcome studies.

In addition to source heterogeneity, the species distribution of *Candida* in the ICU has become increasingly important. Although *Candida albicans* remains a frequent isolate, non-*albicans Candida* species contribute substantially to ICU-associated candidiasis and may differ in antifungal susceptibility, epidemiology, and clinical impact [2,5,6]. *Candida auris* is of particular concern because of its multidrug resistance, environmental persistence, transmission potential, and outbreak behavior in healthcare settings [10,11,12]. Therefore, ICU studies evaluating *Candida*-positive cultures should consider not only mortality outcomes but also microbiological sources, species distribution, antifungal treatment patterns, and the distinction between sterile-site and non-sterile-site isolation.

Against this background, we conducted a single-center retrospective cohort study of adult ICU patients with *Candida*-positive ICU cultures. The final patient-level cohort included 349 critically ill adults with *Candida*-positive ICU cultures. We aimed to evaluate clinical characteristics, microbiological sources, species distribution, antifungal treatment patterns, and outcomes. In response to the heterogeneity inherent to *Candida*-positive ICU cultures, we specifically performed source-stratified analyses and compared blood/sterile-site isolation with non-blood/non-sterile sources. We also assessed variables associated with all-cause 28-day mortality and ICU mortality, while avoiding interpretation of *Candida* score as a stand-alone mortality prediction tool.

## 2. Methods

### 2.1. Study Design and Setting

This single-center, retrospective observational cohort study was conducted in the medical intensive care unit of Marmara University Faculty of Medicine, Istanbul, Türkiye. The study included adult critically ill patients with *Candida*-positive ICU cultures identified between 1 October 2022 and 5 September 2025.

The study was designed to evaluate the clinical characteristics, microbiological sources, species distribution, antifungal treatment patterns, and outcomes of ICU patients with *Candida* isolation. Because *Candida*-positive cultures may represent a heterogeneous spectrum ranging from colonization to candidemia or invasive candidiasis, analyses were performed in the overall cohort and in predefined source-based subgroups.

### 2.2. Study Population

Adult patients aged ≥18 years were eligible if *Candida* spp. was isolated from at least one ICU culture specimen during the study period. Eligible culture sources included blood, sterile fluid, urine, lower respiratory tract samples, catheter-tip cultures, wound/skin cultures, and surveillance or colonization-type cultures.

Patients were excluded if the fungal isolate was not a *Candida* species and no concomitant *Candida* isolation was present. Duplicate patient-level entries were excluded to preserve a patient-level analysis rather than an isolate-level or repeated-culture-level analysis. The final cohort included 349 adult ICU patients with at least one *Candida*-positive ICU culture.

The index record was defined at the patient level. Therefore, the primary analysis was conducted according to patients with *Candida*-positive ICU cultures, not according to the total number of isolates or repeated positive cultures.

### 2.3. Definitions

#### 2.3.1. *Candida* Isolation

*Candida* isolation was defined as recovery of *Candida* spp. from at least one ICU culture specimen obtained during ICU care. Culture sources were categorized as blood, sterile fluid, urine, lower respiratory tract, catheter tip, wound/skin, or surveillance/colonization-type cultures.

Because the clinical significance of *Candida* isolation differs substantially by microbiological source, culture source was evaluated descriptively and analytically. In addition, blood/sterile-site isolation was compared with non-blood/non-sterile-site isolation.

#### 2.3.2. *Candida* Infection and *Candida* Colonization

*Candida* infection and *Candida* colonization were interpreted using a source-based framework consistent with guideline-based principles and contemporary ICU fungal infection literature [1,7].

Bloodstream isolation of *Candida* spp. was classified as candidemia and included in the *Candida* infection group. Isolation of *Candida* spp. from normally sterile fluid was also classified as *Candida* infection.

Isolation from non-sterile sites, including urine, lower respiratory tract samples, wound/skin cultures, catheter-tip cultures, and surveillance/colonization-type cultures, was not considered sufficient by itself to define invasive infection. These non-sterile-site isolates were interpreted together with the infection/colonization classification recorded in the dataset.

Respiratory tract isolation was interpreted cautiously because growth of *Candida* from respiratory secretions usually represents airway colonization rather than true *Candida* pneumonia [1]. Similarly, candiduria was interpreted in relation to clinical context because urinary *Candida* isolation frequently reflects colonization, particularly in catheterized or critically ill patients [1].

For analysis, spelling variants in the infection/colonization variable were harmonized. All entries denoting infection were coded as *Candida* infection, and all entries denoting colonization were coded as *Candida* colonization. Because retrospective classification may be vulnerable to misclassification, source-based analyses and blood/sterile-site stratification were performed as complementary analyses.

#### 2.3.3. Blood/Sterile-Site and Non-Blood/Non-Sterile-Site Isolation

For stratified analyses, patients were grouped according to microbiological source. Blood/sterile-site isolation included patients with *Candida* isolated from blood cultures or normally sterile fluid. Non-blood/non-sterile-site isolation included *Candida* isolation from urine, lower respiratory tract samples, surveillance/colonization-type cultures, wound/skin cultures, and catheter-tip cultures.

This stratification was used because candidemia and sterile-site isolation have different diagnostic and therapeutic implications from candiduria, respiratory tract isolation, and surveillance colonization [1,7].

#### 2.3.4. *Candida* Score

The *Candida* score was evaluated as recorded in the dataset. It was analyzed as a continuous variable and as a categorical variable using a threshold of ≥3, corresponding to the practical application of the original cutoff of >2.5 described by León et al. [8].

The *Candida* score was not interpreted as a mortality prediction score. It was originally developed to support early antifungal decision-making in non-neutropenic critically ill patients with *Candida* colonization when candidal infection is suspected [8]. Therefore, in this study, *Candida* score was evaluated as a marker of cumulative *Candida*-associated risk and illness complexity rather than as a stand-alone prognostic mortality tool.

### 2.4. Data Collection and Variables

Clinical, microbiological, treatment, and outcome data were retrospectively extracted from the study dataset. Before analysis, records were checked for eligibility, duplicate patient-level entries, non-*Candida* fungal isolates, and inconsistent categorical coding.

Collected variables included age, sex, body mass index, comorbidities, malignancy, immunosuppressive treatment, corticosteroid exposure, SOFA score at ICU admission, lactate, neutrophil-to-lymphocyte ratio, procalcitonin, septic shock or noradrenaline requirement, mechanical ventilation duration, ICU length of stay, tracheostomy, central venous catheter use, renal replacement therapy including CRRT/hemodialysis, total parenteral nutrition, antibacterial exposure, carbapenem exposure, glycopeptide exposure, *Candida* score, *Candida* species, microbiological source, bacterial co-detection, antifungal treatment, ICU mortality, and 28-day mortality.

Microbiological variables included isolated *Candida* species and culture sources. Species names were reported using standard Latin nomenclature and italicized throughout the manuscript. Bacterial co-detection was evaluated based on bacterial culture positivity recorded in the dataset.

### 2.5. Antifungal Treatment

Antifungal treatment was evaluated as a treatment-exposure variable. Available antifungal data included whether antifungal treatment was administered and the antifungal agent or class used.

Antifungal agents were categorized as echinocandins, azoles, amphotericin B, nystatin/topical therapy, or no antifungal treatment. Antifungal treatment patterns were summarized in the overall cohort and according to isolated *Candida* species.

Detailed treatment-level variables, including timing of antifungal initiation, empiric versus targeted indication, antifungal susceptibility-guided appropriateness, source control, dose adequacy, and treatment duration, were not uniformly available in the retrospective dataset. Therefore, antifungal treatment was not analyzed as a measure of treatment efficacy, and no causal inference was made regarding antifungal treatment and mortality.

### 2.6. Outcomes

The primary outcome was all-cause 28-day mortality. ICU mortality was defined as all-cause death occurring during the ICU stay.

Neither 28-day mortality nor ICU mortality was interpreted as *Candida*-attributable mortality. Both outcomes were evaluated as overall mortality endpoints in critically ill patients with *Candida*-positive ICU cultures.

Secondary outcomes included ICU mortality, microbiological source distribution, *Candida* species distribution, antifungal treatment patterns, and source-stratified outcomes.

### 2.7. Statistical Analysis

Continuous variables were summarized as median and interquartile range [IQR]. Categorical variables were summarized as frequencies and percentages.

Comparisons between survivors and non-survivors were performed according to 28-day mortality status. Continuous variables were compared using the Mann–Whitney U test. Categorical variables were compared using the chi-square test or Fisher’s exact test, as appropriate.

Because *Candida*-positive ICU cultures represent a heterogeneous clinical and microbiological spectrum, source-stratified analyses were performed. Outcomes were compared across microbiological source groups, including blood, sterile fluid, urine, lower respiratory tract, surveillance/colonization-type cultures, catheter-tip cultures, and wound/skin cultures. Additional analyses compared blood/sterile-site isolation with non-blood/non-sterile-site isolation.

Univariable analyses were first performed to evaluate variables associated with 28-day mortality and ICU mortality. Expanded multivariable logistic regression models were then constructed to address potential confounding. Candidate covariates were selected according to clinical relevance and availability in the dataset.

The expanded models included age, SOFA score at ICU admission, lactate, neutrophil-to-lymphocyte ratio, septic shock/noradrenaline requirement, corticosteroid exposure, carbapenem exposure, CRRT/hemodialysis, malignancy, immunosuppressive treatment, *Candida* score, blood/sterile-site source, and antifungal treatment.

Effect estimates were reported as adjusted odds ratios with 95% confidence intervals. Model discrimination was evaluated using the area under the receiver operating characteristic curve. Model calibration was assessed using the Hosmer–Lemeshow goodness-of-fit test.

A two-sided *p* value < 0.05 was considered statistically significant.

### 2.8. Sample Size Consideration

No formal a priori sample size calculation was performed because of the retrospective observational design. All eligible adult ICU patients with *Candida*-positive ICU cultures during the predefined study period were included.

The final cohort included 349 patients. The number of outcome events was considered adequate for parsimonious multivariable modeling. However, because of the retrospective design and the number of covariates included in the expanded models, the multivariable analyses were interpreted as explanatory rather than predictive.

### 2.9. Ethical Considerations

The study was conducted in accordance with the ethical principles of the Declaration of Helsinki. Ethical approval was obtained from the Marmara University Faculty of Medicine Ethics Committee (05.09.2025-25-0767). Because of the retrospective observational design, the requirement for informed consent was waived by the ethics committee. Patient data were anonymized before analysis.

## 3. Results

### 3.1. Study Cohort and Baseline Characteristics

A total of 349 adult ICU patients with *Candida*-positive ICU cultures were included in the final analysis. The median age was 71 years [IQR, 62–82], and 185 patients were male (53.0%). The median SOFA score at ICU admission was 6 [IQR, 4–8], and the median *Candida* score was 2 [IQR, 1–3].

Overall, all-cause 28-day mortality was 59.0% (206/349), and ICU mortality was 65.9% (230/349).

Baseline clinical characteristics according to 28-day mortality are shown in Table 1. Patients who died within 28 days had higher lactate, NLR, procalcitonin, and *Candida* score values than survivors. Corticosteroid exposure, carbapenem exposure, and septic shock/noradrenaline requirement were also more frequent among non-survivors. ICU length of stay and mechanical ventilation duration were longer among survivors; these variables were interpreted as time-dependent descriptive associations rather than baseline risk factors.

### 3.2. Mycological Characteristics and Treatment Exposure

*Candida* colonization was identified in 247 patients (70.8%), whereas *Candida* infection was identified in 102 patients (29.2%). The infection/colonization distribution did not differ significantly according to 28-day mortality status.

A *Candida* score ≥3 was more frequent among non-survivors than survivors (41.7% vs. 25.9%, *p* = 0.002). Any antifungal treatment was administered in 100 patients (28.7%) and did not differ significantly according to 28-day mortality status. Bacterial culture positivity was recorded in 242 patients (69.3%) (Table 2).

### 3.3. Species and Microbiological Source Distribution

The most frequently isolated species was *Candida albicans* (48.4%), followed by *Candida glabrata* (13.8%), *Candida auris* (12.9%), *Candida kefyr* (7.4%), *Candida parapsilosis* (6.3%), and *Candida tropicalis* (5.7%).

The most common microbiological source was urine (42.4%), followed by lower respiratory tract samples (26.4%) and blood cultures (14.9%) (Table 3).

### 3.4. Source-Stratified Outcomes

Outcomes and treatment exposure varied according to microbiological source. Because some source categories were small, source-specific analyses were interpreted descriptively.

Bloodstream *Candida* isolation was associated with higher ICU mortality than non-blood sources (80.8% vs. 63.3%, *p* = 0.014). When blood and sterile-fluid isolates were combined, blood/sterile-site isolation was associated with higher ICU mortality than non-blood/non-sterile-site isolation (79.2% vs. 63.5%, *p* = 0.026). The difference in 28-day mortality was not statistically significant (66.0% vs. 57.8%, *p* = 0.260). Antifungal treatment was substantially more frequent among patients with blood/sterile-site isolation (94.3% vs. 16.9%, *p* < 0.001) (Table 4).

### 3.5. Exploratory Subgroup Analyses

In unadjusted threshold-based analysis, *Candida* score ≥ 3 was associated with higher 28-day mortality than *Candida* score < 3 (69.9% vs. 53.1%; OR 2.05, 95% CI 1.29–3.27; *p* = 0.002). ICU mortality was also higher among patients with *Candida* score ≥ 3 (74.8% vs. 61.1%; OR 1.89, 95% CI 1.16–3.08; *p* = 0.010). However, this association was attenuated after broader multivariable adjustment.

*Candida auris* was isolated in 45 patients (12.9%). ICU mortality was higher among patients with *Candida auris* than among those with non-*auris Candida* isolates (82.2% vs. 63.5%; OR 2.66, 95% CI 1.20–5.91; *p* = 0.013). The difference in 28-day mortality was not statistically significant. These subgroup findings were considered exploratory.

### 3.6. Antifungal Treatment Patterns According to Species

Overall, 100 patients (28.7%) received antifungal treatment. Echinocandins were the most frequently used systemic antifungal class (62/349, 17.8%), followed by azoles (34/349, 9.7%), amphotericin B formulations (3/349, 0.9%), and nystatin/topical therapy (1/349, 0.3%).

Among patients with *Candida auris* isolation, 24 of 45 patients (53.3%) received antifungal treatment. Echinocandins were the most frequently used systemic antifungal agents in this subgroup (17/45, 37.8%). Because antifungal susceptibility results, treatment timing, source control, and treatment duration were not uniformly available, comparative antifungal effectiveness was not evaluated (Table 5).

### 3.7. Bacterial Co-Detection

Bacterial culture positivity was recorded in 242 patients (69.3%). Bacterial culture positivity was similar between 28-day non-survivors and survivors (70.4% vs. 67.8%, *p* = 0.611), but it was more frequent among ICU non-survivors than ICU survivors in univariable comparison (73.0% vs. 62.2%, *p* = 0.037). However, bacterial culture positivity was not included as an independent predictor in the final expanded models after accounting for inflammatory burden, organ support, antimicrobial exposure, and microbiological source.

A specific bacterial pathogen was recorded in 190 patients. The most frequent recorded bacterial pathogens were *Acinetobacter baumannii*, *Pseudomonas aeruginosa*, *Klebsiella pneumoniae*, and *Enterococcus faecium*. Detailed bacterial pathogen distribution and exploratory *Candida*–bacterial pathogen pair analyses are provided in Supplementary Tables S3 and S4.

### 3.8. Expanded Multivariable Analysis

Expanded multivariable logistic regression models were constructed for all-cause 28-day mortality and ICU mortality. Candidate variables included age, SOFA score at ICU admission, lactate, NLR, septic shock/noradrenaline requirement, corticosteroid exposure, carbapenem exposure, CRRT/hemodialysis, malignancy, immunosuppressive treatment, *Candida* score, blood/sterile-site source, and antifungal treatment.

In the 28-day mortality model, lactate, NLR, and carbapenem exposure were independently associated with mortality. *Candida* score was not independently associated with 28-day mortality after broader adjustment.

In the ICU mortality model, lactate and CRRT/hemodialysis were independently associated with ICU mortality. *Candida* score, blood/sterile-site source, and antifungal treatment were not independently associated with ICU mortality after adjustment (Table 6).

## 4. Discussion

In this single-center retrospective cohort of 349 critically ill adults with *Candida*-positive ICU cultures, we observed several clinically relevant findings. First, *Candida*-positive ICU cultures represented a heterogeneous clinical and microbiological spectrum rather than a single disease entity. Urine, lower respiratory tract, blood, surveillance/colonization-type cultures, catheter-tip cultures, wound/skin cultures, and sterile-fluid isolates differed substantially in their clinical interpretation, infection classification, antifungal treatment exposure, and mortality patterns. Second, blood/sterile-site isolation was associated with higher ICU mortality, although it was not independently associated with mortality after broader adjustment. Third, although *Candida* score was associated with mortality in unadjusted and threshold-based analyses, this association was attenuated after adjustment for inflammatory burden, antimicrobial exposure, organ support, microbiological source, and treatment exposure. Finally, *Candida auris* isolation and bacterial co-detection patterns identified clinically important subgroups, but these findings should be interpreted as exploratory.

The heterogeneity of *Candida*-positive ICU cultures is central to the interpretation of our findings. A positive blood culture for *Candida* spp. has a fundamentally different clinical meaning from candiduria, respiratory tract isolation, catheter-tip growth, or surveillance colonization. Current guideline-based approaches emphasize that respiratory isolation of *Candida* usually reflects colonization rather than true *Candida* pneumonia, and that candiduria frequently represents colonization, particularly in catheterized or critically ill patients [1]. Similarly, the FUNDICU 2024 consensus definitions highlight the need for standardized research definitions and source-specific interpretation of invasive fungal diseases in non-neutropenic adult ICU patients [7]. For this reason, our analysis avoided treating all *Candida*-positive cultures as equivalent disease states and incorporated source-stratified and blood/sterile-site analyses.

The high mortality observed in our cohort is consistent with the known burden of fungal disease in critical care. In the EUCANDICU project, ICU-acquired invasive candidiasis was associated with substantial 30-day mortality, and Al-Dorzi et al. similarly reported high mortality among critically ill patients with invasive candidiasis [5,6]. However, direct comparison with these studies should be made cautiously. Our cohort was not restricted to proven invasive candidiasis or candidemia; instead, it included a broader real-world ICU population with *Candida*-positive cultures from multiple microbiological sources. This broader inclusion likely explains why some source groups, such as urine and respiratory tract cultures, showed different clinical implications from bloodstream or sterile-site isolation.

Blood/sterile-site isolation was associated with higher ICU mortality in source-stratified analysis. This finding is clinically plausible because bloodstream or sterile-site recovery of *Candida* is more likely to represent invasive disease than non-sterile-site isolation. However, blood/sterile-site isolation was not independently associated with mortality after broader adjustment. This suggests that the adverse outcomes observed in these patients may reflect not only microbiological source but also accompanying severity of illness, inflammatory burden, organ support, antimicrobial exposure, and treatment-selection factors. Therefore, microbiological sources should be interpreted as part of an integrated clinical assessment rather than as an isolated prognostic variable.

The interpretation of *Candida* score requires particular caution. The *Candida* score was originally developed to support early antifungal decision-making in non-neutropenic critically ill patients with *Candida* colonization when candidal infection is suspected [8]. It was not designed as a mortality prediction score. In our cohort, *Candida* score ≥3 was associated with higher 28-day and ICU mortality in unadjusted analyses. However, after expanded adjustment, *Candida* score was no longer independently associated with either mortality endpoint. These findings suggest that *Candida* score may reflect cumulative risk, colonization burden, and illness complexity, but should not be interpreted as a stand-alone mortality prediction tool.

This interpretation is consistent with previous evidence showing that colonization-based risk stratification does not automatically translate into improved outcome prediction or treatment benefit. The EMPIRICUS trial showed that empirical micafungin reduced new invasive fungal infections but did not improve 28-day fungal infection-free survival in colonized critically ill patients with ICU-acquired sepsis and multiple organ failure [9]. Similarly, a systematic review and meta-analysis by Alenazy et al. showed that *Candida* colonization was associated with invasive candidiasis risk in non-neutropenic ICU patients with sepsis, but the authors emphasized that available data did not support indiscriminate empirical antifungal treatment in septic patients without documented colonization [13]. These data support our cautious interpretation of *Candida* score as a marker of risk phenotype rather than a definitive mortality predictor.

Inflammatory burden and treatment-related variables were more consistently associated with mortality in the expanded models. Lactate was independently associated with both 28-day and ICU mortality, NLR was independently associated with 28-day mortality, and CRRT/hemodialysis was independently associated with ICU mortality. Carbapenem exposure was also independently associated with 28-day mortality. These associations should not be interpreted as causal effects. Rather, they likely reflect severity of illness, antimicrobial pressure, organ dysfunction, and the complex ICU ecology in which *Candida* isolation occurs. Prior ICU-focused literature has similarly emphasized the changing epidemiology of invasive fungal infections, the role of antimicrobial pressure, the emergence of resistance, and the need for better risk stratification to target antifungal therapy more appropriately [3,14].

The relationship between antifungal treatment and outcome should also be interpreted cautiously. Antifungal exposure differed markedly by source, being far more frequent among patients with blood/sterile-site isolation than among those with non-blood/non-sterile-site isolation. This pattern is clinically expected, but it introduces confounding by indication: patients receiving antifungal therapy are often those with more severe illness, more convincing evidence of invasive disease, or higher perceived risk. In the present study, antifungal treatment was not independently associated with either 28-day or ICU mortality after expanded adjustment. Because detailed treatment-level variables, including timing of initiation, empiric versus targeted indication, antifungal susceptibility-guided appropriateness, source control, dose adequacy, and treatment duration, were not uniformly available, antifungal treatment should be interpreted as a treatment-exposure variable rather than as a measure of treatment efficacy.

*Candida auris* represented a clinically relevant subgroup in our cohort. ICU mortality was higher among patients with *C. auris* isolation than among those with non-*auris Candida* isolates, whereas the difference in 28-day mortality was not statistically significant. This finding is biologically and epidemiologically plausible, given the recognized multidrug resistance, environmental persistence, transmission potential, outbreak behavior, and infection-control challenges associated with *C. auris* in healthcare settings [10,11,12,15]. The WHO fungal priority pathogens list also classifies *C. auris* and *C. albicans* among critical-priority fungal pathogens, underscoring their public health importance and the need for improved surveillance, diagnostics, and antifungal strategies [16]. Nevertheless, the association observed in our cohort should not be interpreted as a pure species effect. *C. auris* isolation may also identify a broader high-risk ICU phenotype characterized by prolonged hospitalization, invasive support, antimicrobial pressure, and environmental exposure. Because susceptibility results and detailed treatment data were not uniformly available, we did not evaluate comparative antifungal effectiveness in *C. auris* cases.

Bacterial co-detection was common in this cohort. Although bacterial culture positivity was not associated with 28-day mortality, it was associated with ICU mortality in univariable analysis. However, bacterial culture positivity was not included as an independent predictor in the final expanded models after accounting for inflammatory burden, organ support, antimicrobial exposure, and microbiological source. Prior studies of mixed *Candida*–bacterial bloodstream infections have shown that these infections occur in clinically severe populations and may be associated with worse outcomes, longer hospitalization, or greater illness complexity [17,18]. In our study, recorded bacterial pathogens and exploratory *Candida*–bacterial pathogen pair analyses were therefore interpreted as descriptive and hypothesis-generating rather than causal.

This study has several strengths. It included a relatively large real-world ICU cohort of adult patients with *Candida*-positive cultures and incorporated clinical, microbiological, treatment, and outcome variables. The analysis explicitly addressed microbiological heterogeneity through source-stratified analyses and blood/sterile-site comparisons. In addition, antifungal treatment patterns were summarized according to isolated *Candida* species, and expanded multivariable models were used to reduce under-adjustment and avoid overinterpreting *Candida* score as an independent mortality predictor. The inclusion of *C. auris*, bacterial co-detection, and species-specific treatment exposure provides a broader view of the ICU ecology surrounding *Candida* isolation.

This study also has important limitations. First, its retrospective single-center design limits causal inference and generalizability. Second, classification of *Candida* infection and colonization may be vulnerable to misclassification, particularly for non-sterile sources such as urine, respiratory tract samples, wound/skin cultures, and catheter-tip cultures. To reduce this limitation, source-stratified and blood/sterile-site analyses were performed. Third, detailed antifungal treatment variables, including timing of initiation, empiric versus targeted indication, source control, treatment duration, and susceptibility-guided appropriateness, were not uniformly available. Fourth, antifungal susceptibility test results were not consistently available in the dataset, limiting interpretation of species-specific treatment adequacy, especially for *C. auris*. Fifth, some subgroup analyses, including sterile-fluid isolates, catheter-tip cultures, wound/skin cultures, individual species groups, and *Candida*–bacterial pathogen pairs, included small numbers and should be interpreted as exploratory. Finally, both 28-day mortality and ICU mortality were all-cause mortality endpoints and should not be interpreted as *Candida*-attributable mortality.

Overall, our findings support a source-specific and clinically integrated approach to *Candida* isolation in the ICU. *Candida*-positive ICU cultures should not be interpreted as a single homogeneous entity. Blood/sterile-site isolation, non-sterile-site colonization, species distribution, bacterial co-detection, inflammatory burden, organ support, and antifungal exposure should be evaluated together. *Candida* score may help identify a higher-risk clinical phenotype, but it should not be used as a stand-alone mortality prediction tool.

## 5. Conclusions

In this retrospective cohort of critically ill adults, *Candida*-positive ICU cultures represented a heterogeneous clinical and microbiological spectrum rather than a single disease entity. Blood/sterile-site isolation was associated with higher ICU mortality, whereas non-blood/non-sterile-site isolates required more cautious interpretation because candiduria, respiratory tract isolation, catheter-tip growth, wound/skin cultures, and surveillance cultures may reflect colonization rather than invasive disease.

Although *Candida* score was associated with mortality in unadjusted analyses, its independent association was attenuated after broader adjustment. Therefore, *Candida* score should be interpreted as a marker of cumulative risk and illness complexity rather than as a stand-alone mortality prediction tool. These findings support a source-specific, clinically integrated, and stewardship-oriented approach to *Candida* isolation in the ICU.

## Figures and Tables

**Table 1:** Baseline clinical characteristics according to 28-day mortality.

Variable	All Patients (n = 349)	Survived (n = 143)	Deceased (n = 206)	*p* Value
Age, years, median [IQR]	71 [62–82]	71 [61–81]	71.5 [62.25–82.75]	0.405
Male sex, n (%)	185 (53.0)	67 (46.9)	118 (57.3)	0.055
BMI, kg/m^2^, median [IQR]	24.5 [22.5–26.4]	24.5 [22.5–27.17]	24.46 [22.5–25.6]	0.304
SOFA score at ICU admission, median [IQR]	6 [4–8]	6 [4–8]	6 [4–8]	0.771
ICU length of stay, days, median [IQR]	13 [7–26]	14 [8.5–32]	13 [6–20.75]	0.007
Mechanical ventilation duration, days, median [IQR]	9 [5–17]	12 [6–23]	7 [5–13]	<0.001
Lactate, median [IQR]	2.1 [1.5–3.3]	1.8 [1.15–2.4]	2.6 [1.8–4.0]	<0.001
NLR, median [IQR]	15.0 [7.0–28.23]	13.4 [6.0–21.66]	16.89 [7.81–34.0]	0.011
Procalcitonin, median [IQR]	1.0 [0.3–3.31]	0.61 [0.2–2.3]	1.27 [0.45–4.42]	<0.001
*Candida* score, median [IQR]	2 [1–3]	2 [1–3]	2 [2,3]	<0.001
Corticosteroid use, n (%)	214 (61.3)	78 (54.5)	136 (66.0)	0.030
Carbapenem exposure, n (%)	281 (80.5)	101 (70.6)	180 (87.4)	<0.001
Septic shock/noradrenaline requirement, n (%)	229 (65.6)	80 (55.9)	149 (72.3)	0.002
CRRT/hemodialysis, n (%)	144 (41.3)	52 (36.4)	92 (44.7)	0.134
Malignancy, n (%)	105 (30.1)	39 (27.3)	66 (32.0)	0.340
Immunosuppressive treatment, n (%)	134 (38.4)	50 (35.0)	84 (40.8)	0.294

Abbreviations: BMI, body mass index; CRRT, continuous renal replacement therapy; ICU, intensive care unit; IQR, interquartile range; NLR, neutrophil-to-lymphocyte ratio; SOFA, Sequential Organ Failure Assessment.

**Table 2:** Mycological and treatment characteristics according to 28-day mortality.

Variable	All Patients (n = 349)	Survived (n = 143)	Deceased (n = 206)	*p* Value
*Candida* colonization, n (%)	247 (70.8)	106 (74.1)	141 (68.4)	0.251
*Candida* infection, n (%)	102 (29.2)	37 (25.9)	65 (31.6)	0.251
*Candida* score ≥ 3, n (%)	123 (35.2)	37 (25.9)	86 (41.7)	0.002
Any antifungal treatment, n (%)	100 (28.7)	37 (25.9)	63 (30.6)	0.339
Bacterial culture positivity, n (%)	242 (69.3)	97 (67.8)	145 (70.4)	0.611
Blood/sterile-site source, n (%)	53 (15.2)	18 (12.6)	35 (17.0)	0.260

**Table 3:** Distribution of *Candida* species and microbiological sources.

Species	n (%)
*Candida albicans*	169 (48.4)
*Candida glabrata*	48 (13.8)
*Candida auris*	45 (12.9)
*Candida kefyr*	26 (7.4)
*Candida parapsilosis*	22 (6.3)
*Candida tropicalis*	20 (5.7)
*Candida lusitaniae*	6 (1.7)
*Candida krusei*	5 (1.4)
*Candida dubliniensis*	3 (0.9)
*Candida inconspicua*	2 (0.6)
*Candida norvegensis*	2 (0.6)
*Candida guilliermondii*	1 (0.3)
Microbiological source	n (%)
Urine	148 (42.4)
Lower respiratory tract	92 (26.4)
Blood	52 (14.9)
Surveillance/colonization-type culture	39 (11.2)
Wound/skin	12 (3.4)
Catheter tip	5 (1.4)
Sterile fluid	1 (0.3)

**Table 4:** Outcomes according to microbiological source.

Source	n	*Candida* Infection	28-Day Mortality	ICU Mortality	Antifungal Treatment	*Candida* Score, Median [IQR]
Blood	52	52/52 (100.0)	35/52 (67.3)	42/52 (80.8)	49/52 (94.2)	2 [1–3]
Sterile fluid	1	1/1 (100.0)	0/1 (0.0)	0/1 (0.0)	1/1 (100.0)	4 [4–4]
Urine	148	10/148 (6.8)	92/148 (62.2)	94/148 (63.5)	11/148 (7.4)	2 [1–3]
Lower respiratory tract	92	28/92 (30.4)	55/92 (59.8)	63/92 (68.5)	28/92 (30.4)	2 [1–3]
Surveillance/colonization-type culture	39	0/39 (0.0)	16/39 (41.0)	20/39 (51.3)	0/39 (0.0)	3 [1.5–3]
Catheter tip	5	4/5 (80.0)	1/5 (20.0)	3/5 (60.0)	4/5 (80.0)	2 [2–2]
Wound/skin	12	7/12 (58.3)	7/12 (58.3)	8/12 (66.7)	7/12 (58.3)	2.5 [2–4]
**Source group**		**n**	**28-day mortality**		**ICU mortality**	**Antifungal treatment**
Blood/sterile-site source		53	35/53 (66.0)		42/53 (79.2)	50/53 (94.3)
Non-blood/non-sterile-site source		296	171/296 (57.8)		188/296 (63.5)	50/296 (16.9)
*p* value			0.260		0.026	<0.001

**Table 5:** Antifungal treatment according to isolated *Candida* species.

Species	n	No Antifungal Treatment	Echinocandin	Azole	Amphotericin B	Nystatin/Topical
*Candida albicans*	169	134 (79.3)	21 (12.4)	13 (7.7)	1 (0.6)	0 (0.0)
*Candida glabrata*	48	36 (75.0)	7 (14.6)	4 (8.3)	1 (2.1)	0 (0.0)
*Candida auris*	45	21 (46.7)	17 (37.8)	6 (13.3)	0 (0.0)	1 (2.2)
*Candida kefyr*	26	15 (57.7)	5 (19.2)	6 (23.1)	0 (0.0)	0 (0.0)
*Candida parapsilosis*	22	12 (54.5)	7 (31.8)	3 (13.6)	0 (0.0)	0 (0.0)
*Candida tropicalis*	20	16 (80.0)	4 (20.0)	0 (0.0)	0 (0.0)	0 (0.0)
Other *Candida* spp.	19	15 (78.9)	1 (5.3)	2 (10.5)	1 (5.3)	0 (0.0)

**Table 6:** Expanded multivariable logistic regression models for mortality outcomes.

Variable	28-Day Mortality, Adjusted OR (95% CI)	*p* Value	ICU Mortality, Adjusted OR (95% CI)	*p* Value
Age	1.01 (0.99–1.02)	0.245	1.00 (0.99–1.02)	0.707
SOFA score at ICU admission	1.00 (0.93–1.06)	0.927	0.99 (0.93–1.06)	0.810
Lactate	1.19 (1.06–1.35)	0.005	1.29 (1.11–1.51)	0.001
NLR	1.02 (1.01–1.03)	0.007	1.01 (1.00–1.03)	0.072
Septic shock/noradrenaline requirement	1.24 (0.69–2.24)	0.464	1.37 (0.75–2.50)	0.312
Corticosteroid exposure	1.47 (0.87–2.51)	0.153	1.21 (0.69–2.11)	0.507
Carbapenem exposure	2.30 (1.23–4.32)	0.009	1.74 (0.92–3.29)	0.086
CRRT/hemodialysis	0.98 (0.59–1.62)	0.930	1.84 (1.08–3.15)	0.026
Malignancy	1.02 (0.58–1.79)	0.945	1.56 (0.85–2.88)	0.152
Immunosuppressive treatment	1.26 (0.71–2.24)	0.425	0.98 (0.53–1.80)	0.943
*Candida* score	1.21 (0.95–1.55)	0.123	1.12 (0.87–1.45)	0.387
Blood/sterile-site source	1.03 (0.44–2.42)	0.938	1.12 (0.43–2.90)	0.823
Antifungal treatment	1.02 (0.52–1.98)	0.953	1.62 (0.78–3.38)	0.196

Model performance: 28-day mortality model: n = 344; events = 204; AUC = 0.730; Nagelkerke R^2^ = 0.181; Hosmer–Lemeshow *p* = 0.407. ICU mortality model: n = 344; events = 228; AUC = 0.750; Nagelkerke R^2^ = 0.206; Hosmer–Lemeshow *p* = 0.124. Abbreviations: AUC, area under the receiver operating characteristic curve; CI, confidence interval; CRRT, continuous renal replacement therapy; NLR, neutrophil-to-lymphocyte ratio; OR, odds ratio; SOFA, Sequential Organ Failure Assessment.

## Data Availability

The datasets used and/or analyzed during the current study are available from the corresponding author on reasonable request.

## Supplementary Materials

The following supporting information can be downloaded at: https://www.mdpi.com/article/10.3390/jcm15103710/s1, Table S1: Full baseline clinical characteristics according to 28-day mortality; Table S2: Clinical and microbiological characteristics according to *Candida* colonization versus infection; Table S3: Recorded bacterial pathogens in the cohort; Table S4: Exploratory *Candida*–bacterial pathogen co-detection pairs among patients classified as *Candida* infection.
