# Supplementary material for: Clinical Characteristics, Microbiological Sources, and Outcomes of Candida-Positive ICU Cultures in Critically Ill Adults
Source: J Clin Med. 2026 May 12;15(10):3710. doi: 10.3390/jcm15103710 (PMC13207584; doi:10.3390/jcm15103710)
Supplement: Supplementary file 1 [file jcm-15-03710-s001.zip › jcm-4294362-supplementary.pdf]

Supplementary Table S1. Full baseline clinical characteristics according to 28-day mortality

**Variable**	**All patients (n=349)**	**Survived (n=143)**	**Deceased (n=206)**	**p value**
Chronic kidney disease, n (%)	60 (17.2)	24 (16.8)	36 (17.5)	0.866
COPD/chronic lung disease, n (%)	88 (25.2)	32 (22.4)	56 (27.2)	0.309
Neurological disease, n (%)	105 (30.1)	48 (33.6)	57 (27.7)	0.238
Hematological malignancy, n (%)	38 (10.9)	13 (9.1)	25 (12.1)	0.369
Solid tumor, n (%)	67 (19.2)	23 (16.1)	44 (21.4)	0.218
Neutropenia on admission, n (%)	16 (4.6)	3 (2.1)	13 (6.3)	0.064
Surgery in previous 3 months, n (%)	85 (24.4)	37 (25.9)	48 (23.3)	0.582
Tracheostomy, n (%)	54 (15.5)	30 (21.0)	24 (11.7)	0.018
Central venous catheter, n (%)	224 (64.2)	86 (60.1)	138 (67.0)	0.189
Foley catheter, n (%)	328 (94.0)	135 (94.4)	193 (93.7)	0.782
Total parenteral nutrition, n (%)	35 (10.0)	19 (13.3)	16 (7.8)	0.091
Glycopeptide exposure, n (%)	202 (57.9)	76 (53.1)	126 (61.2)	0.136
Other antibiotic exposure, n (%)	326 (93.4)	133 (93.0)	193 (93.7)	0.801
CRP, median [IQR]	101.2 [47–171.3]	82.38 [44.19–161.3]	109.96 [50.25–178.22]	0.140
BUN, median [IQR]	35 [20–59]	31 [19–48.5]	39 [21–67.75]	0.013
Creatinine, median [IQR]	1.1 [0.66–1.96]	1.03 [0.63–1.88]	1.17 [0.67–2]	0.277
AST, median [IQR]	34.9 [21–70.7]	27.06 [17–45.35]	43.9 [26.2–99.5]	<0.001
ALT, median [IQR]	23 [12–47]	18 [9.43–32.9]	27.55 [13.6–59.17]	<0.001

Abbreviations: ALT, alanine aminotransferase; AST, aspartate aminotransferase; BMI, body mass index; BUN, blood urea nitrogen; COPD, chronic obstructive pulmonary disease; CRP, C-reactive protein; CRRT, continuous renal replacement therapy; CVVHD, continuous veno-venous hemodialysis; ICU, intensive care unit; IQR, interquartile range; NLR, neutrophil-to-lymphocyte ratio; SOFA, Sequential Organ Failure Assessment.

Supplementary Table S2. Clinical and microbiological characteristics according to *Candida* colonization versus infection

**Variable**	***Candida* colonization (n=247)**	***Candida* infection (n=102)**	**p value**
Age, years, median [IQR]	73 [64–83]	67 [56.25–77]	0.001
ICU length of stay, days, median [IQR]	12 [7–23]	16 [8–33.5]	0.075
Mechanical ventilation duration, days, median [IQR]	9 [5–16.5]	9 [6–17]	0.813
SOFA score at ICU admission, median [IQR]	6 [4–8]	6 [4–8.75]	0.352
Lactate, median [IQR]	2 [1.4–3.2]	2.4 [1.7–4]	0.055
NLR, median [IQR]	14.25 [7.7–26.44]	16.21 [5.3–31]	0.971
Procalcitonin, median [IQR]	0.94 [0.29–2.44]	1.17 [0.31–9.25]	0.039
Candida score, median [IQR]	2 [1–3]	2.5 [2–3]	<0.001
Blood/sterile-site source, n (%)	0 (0.0)	53 (52.0)	<0.001
Any antifungal treatment, n (%)	2 (0.8)	98 (96.1)	<0.001
Bacterial culture positivity, n (%)	161 (65.2)	81 (79.4)	0.009
Central venous catheter, n (%)	151 (61.1)	73 (71.6)	0.064
CRRT/hemodialysis/CVVHD, n (%)	96 (38.9)	48 (47.1)	0.157
Carbapenem exposure, n (%)	195 (78.9)	86 (84.3)	0.250
Glycopeptide exposure, n (%)	130 (52.6)	72 (70.6)	0.002

**Variable**	***Candida* colonization (n=247)**	***Candida* infection (n=102)**	**p value**
Septic shock/noradrenaline requirement, n (%)	158 (64.0)	71 (69.6)	0.313
28-day mortality, n (%)	141 (57.1)	65 (63.7)	0.251
ICU mortality, n (%)	151 (61.1)	79 (77.5)	0.003

Interpretation note: This comparison is descriptive. Because infection/colonization classification was retrospective and source-dependent, source-stratified analyses were used as complementary analyses in the main manuscript.

Supplementary Table S3. Recorded bacterial pathogens in the cohort

A specific bacterial pathogen was recorded in 190 patients.

**Bacterial pathogen**	**n**	**% of total cohort (n=349)**	**% among recorded bacterial pathogens (n=190)**
Acinetobacter baumannii	38	10.9	20.0
Pseudomonas aeruginosa	33	9.5	17.4
Klebsiella pneumoniae	29	8.3	15.3
Enterococcus faecium	22	6.3	11.6
Escherichia coli	16	4.6	8.4
Stenotrophomonas maltophilia	14	4.0	7.4
Staphylococcus aureus	12	3.4	6.3
Enterococcus faecalis	12	3.4	6.3
Serratia marcescens	9	2.6	4.7
Proteus mirabilis	5	1.4	2.6

Supplementary Table S4. Exploratory *Candida*–bacteria co-detection pairs among patients classified as *Candida* infection

Among 102 patients classified as *Candida* infection, a recorded bacterial pathogen was present in 65 patients.

***Candida* species**	**Bacterial pathogen**	**n**
Candida albicans	*Acinetobacter baumannii*	5
Candida albicans	*Staphylococcus aureus*	4
Candida auris	*Klebsiella pneumoniae*	4
Candida auris	*Enterococcus faecalis*	3
Candida auris	*Escherichia coli*	3
Candida albicans	*Pseudomonas aeruginosa*	3
Candida albicans	*Stenotrophomonas maltophilia*	3
Candida albicans	*Escherichia coli*	3
Candida tropicalis	*Enterococcus faecium*	2
Candida albicans	*Proteus mirabilis*	2
Candida auris	*Acinetobacter baumannii*	2
Candida parapsilosis	*Pseudomonas aeruginosa*	2
Candida glabrata	*Klebsiella pneumoniae*	2
Candida glabrata	*Acinetobacter baumannii*	2
Candida albicans	*Enterococcus faecium*	2
Candida auris	*Pseudomonas aeruginosa*	2
Candida glabrata	*Enterococcus faecium*	2
Candida kefyr	*Serratia marcescens*	2

***Candida* species**	**Bacterial pathogen**	**n**
Candida auris	*Proteus mirabilis*	2
Candida auris	*Enterococcus faecium*	2
